# Determinants of Technical Efficiency in Public Hospitals: The Case of Saudi Arabia

**DOI:** 10.1186/s13561-020-00282-z

**Published:** 2020-08-01

**Authors:** Ahmed D. Alatawi, Louis W. Niessen, Jahangir A. M. Khan

**Affiliations:** 1 Health Economics Group, Department of Clinical Sciences, Liverpool School of Tropical Medicine, LSTM, Room 1966-215, Pembroke Place, Liverpool, L3 5QA UK; 2 Department of Clinical Pharmacy, Pharmacy College, University of Al-Jouf, Sakaka, Saudi Arabia; 3 Department of International Public Health, Health Economics Group, Liverpool School of Tropical Medicine, Room 1966-215, Pembroke Place, Liverpool, L3 5QA UK; 4 Division of Health Sciences, University of Warwick, Coventry, UK; 5 Department of Learning, Informatics, Management and Ethics, Karolinska Institute, Stockholm, Sweden

**Keywords:** Technical efficiency, Public hospitals, Healthcare utilization, Environmental factors, Population characteristics, Saudi Arabia

## Abstract

**Objective:**

In this study, we investigate the effect of the external environmental and institutional factors on the efficiency and the performance of the public hospitals affiliated to the Ministry of Health (MOH) in the Kingdom of Saudi Arabia (KSA). We estimate the demographic and socioeconomic characteristics of catchment populations that explain the demand for health services.

**Methods:**

We apply descriptive analysis to explore what external factors (demographic and socioeconomic factors) can explain the observed differences in technical efficiency scores. We use Spearman’s rank correlation, multivariate Tobit regression and Two-part model to measure the impact of the explanatory variables (i.e. population density, nationality, gender, age groups, economic status, health status, medical interventions and geographic location) on the efficiency scores.

**Results:**

The analysis shows that the external factors had a significant influence on efficiency scores. We find significant associations between hospitals efficiency scores and number of populations in the catchment area, percentage of children (0–5 years old), the prevalence of infectious diseases, and the number of prescriptions dispensed from hospital’s departments. Also, the scores significantly associate with the number of populations who faced financial hardships during medical treatments, and those received financial support from social administration. That indicates the hospitals that serve more patients in previous characteristics are relatively more technically efficient.

**Conclusions:**

The environmental and institutional factors have a crucial effect on efficiency and performance in public hospitals. In these regards, we suggested improvement of health policies and planning in respect to hospital efficiency and resource allocation, which consider the different demographic, socioeconomic and health status of the catchment populations (e.g., population density, poverty, health indicators and services utilization). The MOH should pay more attention to ensure appropriate allocation mechanisms of health resources and improve utilization of health services among the target populations, for securing efficient and equitable health services.

## Key points

- The demographics and socioeconomic characteristics of catchment populations have a significant effect on efficiency and performance in public hospitals.
- The appropriate allocation mechanisms of health resources and improve utilization of health services among the target populations, are required for securing efficient and equitable health services.
- This is the first research of determinants of technical efficiency consider population characteristics influence the performance of public hospitals in Saudi Arabia.

## Introduction

National policies worldwide aim at effective, efficient, and equitable healthcare systems. Globally, the United Nations have recognized the critical role of health systems and countries subscribed to this in the formulation of the universal health coverage goals (UHC) [1]. The Kingdom of Saudi Arabia (KSA), during the recent decades, has been experiencing considerable population growth, longer life expectancy and increase of lifestyle-related diseases, which increased demand for healthcare services and relevant health expenditures [2–4]. Thus, it is important to ensure the effective utilization of existing resources in the public health system to facilitate meeting the UHC goals.

The KSA government, under article 31 of the national constitution, guarantees free medical care to all citizens in the public sector’s facilities throughout the country [5]. The Ministry of Health (MOH), the main provider of healthcare services in the public sector in KSA, administers 60% of all providers of healthcare [6]. Besides, MOH plans health strategies, formulates health policies, supervises all health service delivery programs, health education, promotion, and monitoring of all health-related activities in KSA [7]. The MOH provides primary, secondary and tertiary healthcare services through 2361 primary healthcare centres and 282 hospitals, including 43,080 beds [7, 8]. Public (MOH-affiliated) hospitals also provide secondary and tertiary health services through general and specialized hospitals located in 20 administrative districts [5].

In the KSA, public spending on health was 67.8% of the country’s total health expenditure, which corresponds to 3.9% of GDP for the year 2016 [9]. Such expenditure has significantly increased by 24.7% from the year 2013 to 2017 [8]. However, MOH health statistics showed considerably lower availability of services, given the high health expenditures in KSA compared with other countries, which indicated inefficiency in utilization of health resources [9].

According to the World Health Report (WHR) in 2010, it is estimated that about 20–40% of all health sector resources are vanished globally due to inefficiency in healthcare systems [10]. Moreover, such loss of health resources due to hospital-related inefficiency was estimated to USD 300 billion annually [10]. Since the hospitals are the main consumers of health resources; the hospital efficiency is crucial to the overall efficiency of any health systems as concluded by a broad range of literature [11, 12]. Therefore, governments are required to conduct efficiency analysis of their health sectors and identify the causes of inefficiency, to undertake necessary policy and practices for ensuring efficient utilization of public health resources [13]. It is essential to reduce the consumption of excessive resources in producing healthcare services and understand the determinates of inefficiency for healthcare systems [14].

However, there is a lack of empirical research on the efficiency assessment of public hospitals and the determinants of inefficiency in general and in the region. A systematic review of public hospital efficiency studies in the Gulf region has shown the number of studies to be very limited, as efficiency analysis is a novel and rarely applied approach in the Gulf region, including the KSA [15]. The review found only two studies conducted in KSA context; a study by Helal and Elimam in 2017, which assessed the efficiency of health services at districts level during the year 2014 in KSA. The average technical efficiency score was 0.92; also, 45% of the districts achieved the efficient score [16]. Another efficiency analysis conducted in 2013 of 20 public hospitals, under private sector management, found that 60% of the study sample had not achieved the efficient score [17].

In a previous study, we applied technical efficiency analysis of 91 public hospitals, affiliated to the MOH in the KSA that provide health services to around seven million persons in the country [18]. We used a non-parametric, Data Envelopment Analysis (DEA), which has for many years, been the most commonly used technique for measuring the technical efficiency in healthcare [12, 19]. The study found that most public hospitals (75.8%) were technically inefficient with the surplus amount (slack) around one-quarter of the total health resources used in the hospitals [18]. Also, the efficiency level differs based on hospital size and their geographic locations and reallocation of health resources appeared to be important to facilitate the optimal use of medical capacity [18].

Efficiency literatures emphasized that the performance of public hospitals is influenced not only by the internal factors but also by external factors beyond the control of hospital management, which may have an impact on technical efficiency, for instance, Kontodimopoulos et al. 2007; Mitropoulos et al. 2016; Ahmed et al. 2019 and Cheng et al. 2015 [14, 20–22]. Although these external factors are not used to construct the frontier analysis (e.g. DEA analysis), their influence on efficiency measurement needs to be investigated [21]. More specifically, these external factors are represented by environmental variables that are related to management, demographic characteristics of the catchment area of the hospitals, and organizational structure [23]. Therefore, in the last few years several efficiency studies have focused in analyzing the determinants of inefficiency, for instance, Ahmed et al. 2019, Gok and Sezen, 2013, Cheng et al. 2015 and Kontodimopoulos et al. 2007 [14, 20, 22, 24]. The effects of the determinants on the inefficiency scores were estimated in those studies.

In this current study, we investigated empirically which external factors that may affect the efficiency and productivity of the MOH public hospitals in the KSA. It means that we intended to provide a better explanation of the variations in efficiency levels of 91 public hospitals in the KSA [18]. In addition, we highlighted which environmental characteristics and organizational factors influenced the efficiency based on the demand for health services. This research facilitates in generating useful information for designing the health policies and reforms to achieving improvement towards optimal use of health resources in the public hospitals.

## Method

### Data sources and variables

The focus of the current analysis is to assess the variation of public hospitals efficiency levels and to what degree the differences in the efficiency scores can be explained by observed external factors (demand factors) such as health status, demographic and socioeconomic characteristics of the populations in the catchment area of each hospital. In other words, we analyzed the factors that influence healthcare utilization with respect to the demographic and socioeconomic structure of the population variables in the catchment area of each hospital that predict the efficiency scores. Beforehand, we estimated technical efficiency scores of the 91 public hospitals, affiliated to the MOH in the KSA using Data Envelopment Analysis (DEA) technique (Table 1) [18].

**Table 1:** Technical efficiency scores of the public hospitals categorized by hospital size and geographic location

Hospital category	Number of hospitals	Average CRS Score (SD)	Average VRS Score (SD)	Average Scale Score (SD)
All Public hospitals	91	0.76 (0.23)	0.87 (0.18)	0.87 (0.18)
***Hospitals categorized by size***				
Large hospitals > = 500 beds	8	0.65 (0.27)	0.75 (0.3)	0.87 (0.13)
Upper-medium hospitals: 300–499 beds	22	0.76 (0.19)	0.80 (0.19)	0.94 (0.07)
Lower-medium hospitals: 200–299 beds	22	0.73 (0.25)	0.79 (0.19)	0.90 (0.18)
Small hospitals: < 200 beds	39	0.79 (0.23)	0.96 (0.09)	0.82 (0.22)
***Hospitals categorized by geographic location***				
South region	22	0.75 (0.25)	0.89 (0.18)	0.83 (0.23)
East region	8	0.80 (0.28)	0.85 (0.23)	0.90 (0.16)
North region	17	0.75 (0.28)	0.84 (0.23)	0.90 (0.2)
Central region	24	0.83 (0.18)	0.89 (0.16)	0.93 (0.10)
West region	20	0.68 (0.2)	0.85 (0.17)	0.81 (0.17)

Source: Alatawi et al., 2020 [18]

However, external variables, i.e. environmental and institutional factors, which were not under the control of the public hospital efficiency analysis, need to be considered in an additional evaluation since such factors are a potential source of inefficiency [21, 25]. External variables are usually included in a second phase of the analysis in order to explain the reasons whether the public hospital is inefficient [26]. The efficiency evaluation of a public hospital should explicitly include external factors; however, it could be argued that if such variables were not included in the estimation of technical efficiency, the results obtained would not be operationally valid [25].

The external variables have been selected based on a review of literature on efficiency analysis of public hospitals, and the effect of these factors on the production of healthcare services [14, 22, 26]. Factors that affect the efficiency of public hospitals were classified to institutional factors, i.e. hospital size/number of beds and environmental factors, i.e. demographics of the catchment population, socioeconomics, e.g. poverty indicators (financial hardship) and health status, e.g. chronic and infectious diseases cases and related treatment activate [22, 26].

The following environmental factors including demographic and socioeconomic factors were chosen: (1) number of population in the hospital’s catchment area (registered in the selected public hospital); (2) percentage of Saudi and non-Saudi population; (3) proportion of male and female; (4) percentage of 0–5 years old children, percentage of 5–15 years old children, percentage of 15–45 years old population, percentage of 45–65 years old, and percentage of the elderly population with more than 65 years old; (5) the number of populations who faced financial hardship during the treatment and required hospitalization for more than 3 months due to financial causes (indicator of poverty in the hospital area); (6) number of cases investigated and supported by the public social administration for economic reasons within the hospital area (indicator of poverty in the hospital area); (7) number of Infectious and parasitic disease cases, and the number of chronic disease cases (e.g. diabetes and cardiovascular disease) that were treated in the hospital; (8) number of dispensed prescriptions from the pharmacy department for treating chronic or infectious disease cases. (9) the geographic location of the hospital, i.e. central, west, east, north and south regions. All data of the external variables and efficiency scores were from the year 2017.

The general hospitals used in the analysis included 21,528 out of 398,68 (54%) of the total active hospital beds provided by the MOH (public sector). We included in the analysis 91 hospitals while six more were excluded, due to missing data. The institutional data of the hospitals and population characteristics in each hospital catchment area for 2017 were collected by the main author from official statistical, informational and research databases of Administration of Statistics and Information and Administration of Research and Studies, which was affiliated to the Ministry of Health MOH in Riyadh city, following approval from the designated authority.

### Data analysis

Descriptive statistics on the hospital efficiency score and the external factors (demand for healthcare) were presented initially, reflecting the mean values and the correlation between the variables (Table 2). Efficiency scores were compared against each of the environmental and institutional variables of the public hospitals, using Spearman’s rank correlation as a non-parametric measure of statistical dependence between two variables [27].

Several ways have been developed to incorporate the effect of external factors into the production process in estimating efficiency scores through DEA. In this context, we found several literatures which regressed the efficiency scores by the environmental variables, applying either Tobit regression or Ordinary Least Square estimation [23, 28].

In this paper, the Tobit regression model was employed to measure the association between the inefficiency scores and explanatory variables (external factors). Since the efficiency scores range between zero and one, and some of the data tend to concentrate on these boundary values (i.e., censored for the DMUs with a value at one), ordinary least squares might be inapplicable in this context [20, 22, 28].

For convenience, in the Tobit model, we assumed a censoring point at zero in the model. As a result, efficient hospitals would have score zero, and the inefficient ones will have a score greater than zero [22]. We transformed the CRS and VRS technical efficiency scores into CRS and VRS inefficiency scores, like Asbu, EZ. In 2000, and left censoring at zero as follows [28, 29]:

Inefficiency score = (1/Technical efficiency score) – 1 (i).

This transformation of the dependent variable (CRS scores), would thus reverse the signs of the coefficient in the regression model [22]. It means that the negative coefficient of a given factor with inefficiency score would reflect a positive coefficient with efficiency scores.

We also applied Two-part model to assess the effect of the explanatory variables on CRS efficiency scores, since we could not adjust for the assumption that the same variables and the same parameters control censorship and non-censorship in the tobit model [30]. In the two-part model, we considered the censorship in the first part of the model and non-censorship in the second part. In the first part, the dependent variable was considered to have a dichotomous nature for explaining the variations between the hospitals with and without full efficiency. In the second part, the variations among the inefficient hospitals were explained by the independent variables.

The CRS technical inefficiency scores were regressed to estimate the association between technical efficiency and selected institutional and environmental characteristics. Data analysis was performed with STATA SE (version 16).

## Results

Descriptive statistics of the selected environmental factors of the public hospitals are shown in Table 2. The total number of populations of the catchment areas of the 91 hospitals are 6,609,215 persons, with an average of 72,629 and standard deviation (SD) of 71,474 per hospital ranging from 1785 to 466,608 in each catchment area. Majority of hospital catchment areas’ population were Saudi citizens (average 90.7% and SD 6.05%), compared with non-Saudis (average 9.3%). The proportion of female was higher than male, with a mean of 55.8% (SD 7.6%) compared with 44.2% for male. Most of the included populations were adults and their age ranged between 45 to 65 years (average 37% and SD 12.97%), followed by 15 to 45 years old (27.8% and SD 14.14%). The proportion of the elderly population (> 65 years) was relatively high (average 20.5%). However, the number of children population were relatively low than any other age groups. Percentage of 0–5 years old children were 7%, and the corresponding proportion of 5–15 years old were 6.9%.

**Table 2:** Descriptive statistics of the environmental factors

Variable	Mean	Std. Dev.	Quartiles		
			25%	50% (Median)	75%
**Population of catchment area (n)**	72,629	71,474	26,865	56,528	89,036
**Saudi (%)**	90.7	6.05	87.53	91.81	95.62
**Non-Saudi (%)**	9.3	6.05	4.38	8.19	12.47
**Male (%)**	44.2	7.6	41.05	44.77	49.51
**Female (%)**	55.8	7.6	50.49	55.23	58.95
**Children 0–5 years (%)**	7.0	7.12	2.20	4.34	8.44
**Children > 5–15 years (%)**	6.9	3.39	4.22	6.65	8.48
**Adults > 15–45 years (%)**	27.8	14.14	18.68	25.36	38.43
**Adults > 45–65 years (%)**	37.0	12.97	28.21	38.82	44.49
**Elderly > 65 years (%)**	20.5	9.89	12.20	19.13	27.39
**Infectious disease (%)**	17.6	14.40	3366	8362	16,707
**Chronic disease cases (%)**	82.4	14.40	27,996	47,119	91,789
**Antimicrobial prescriptions (n)**	480,160	678,679	45,200	156,484	597,543
**Chronic-meds prescriptions (n)**	1,206,603	1,695,256	94,652	326,030	1,790,810
**POP Financial Hardship (n)**	813	1232	215	510	943
**POP social-economic supp. (n)**	45,954.5	6428.9	1198	2206	4249

Moreover, the number of infectious and peracetic disease cases in 2017 on average were 15,590, corresponding to 17.6% of the total registered populations. Registered patients of chronic diseases, e.g. cardiovascular, diabetes and nervous system diseases on average was 72,558 or 82.4% of the total population. Patients received, on average, 480,160 (SD 678,679) antimicrobial prescriptions dispensed from the pharmacy department of the hospitals. They also get the benefits of 1,206,603 (SD 1,695,256) dispensed prescriptions on average for chronic disease medications.

However, around 813 patients in each catchment area faced financial hardship during health treatment and required hospitalization for more than 3 months for economic reasons. The number of cases that were investigated and supported by the public social administration for economic reasons within the hospital catchment area was on average 4595 during the year 2017.

We estimated Spearman rank correlations between inefficiency scores [(1/TE) – 1] of CRS, VRS and scale efficiency and external and environmental variables in Table 3.

**Table 3:** Spearman correlation of Inefficiency association with external variables (*N* = 91)

External factors	CRS	***P***-value	VRS	***P***-value	Scale	***P***-value
Population (n)	−0.3416***	0.0009	− 0.0558	0.5991	− 0.3606***	0.0004
Hospital bed (n)	0.1029	0.332	0.4398***	0.00	−0.1626	0.1236
Non-Saudi (%)	0.048	0.6514	0.097	0.3605	0.0494	0.6419
Female (%)	−0.1869*	0.0761	−0.0464	0.662	−0.2130**	0.0427
Children (0–5 years) (%)	−0.4763***	0.00	−0.2083**	0.0476	−0.5310***	0.00
Adults (15–45 years) (%)	−0.0626	0.5558	0.0603	0.5699	−0.0987	0.3518
Elderly (> 65 years) (%)	0.0936	0.3776	−0.0603	0.5704	0.1271	0.2298
Infectious disease (%)	−0.5521***	0.00	−0.3643***	0.0004	−0.5300***	0.00
Anti-microbial pres. (n)	−0.0876	0.4092	−0.0152	0.8864	−0.0027	0.9797
Chronic dis. Pres. (n)	−0.0764	0.4715	0.0135	0.8991	−0.0275	0.7957
Financial hardship (n)	−0.4694***	0.00	−0.2343**	0.0254	−0.5261***	0.00
Social support (n)	−0.2450**	0.0193	−0.0292	0.7838	−0.3220***	0.0019

CRS, constant return to scale; VRS, variable return to scale; Non-Saudi, percent of non-Saudi; Female, the proportion of female; Infectious disease, percentage of infectious diseases, Anti-microbial pres., amount of antimicrobial dispensed medications; Chronic dis. Pres., chronic medications dispensed; Financial hardship, the number of the population faced financial hardship; Social support, number received benefits of social-economic support. *Significant at the 0.10 level. **Significant at the 0.05 level. ***Significant at the 0.01 level

The analysis of inefficiency estimates against the environmental factors shows that 6 of the 12 factors had a significant correlation at *P* < 0.1, *P* < 0.05 or *P* < 0.01. However, the external variables were more associated with CRS and scale inefficiency compared with VRS inefficiency (4 variables were significant).

The most significant associations were between inefficiency scores and number of populations in the catchment area, percentage of children, the prevalence of infectious diseases, and the number of populations who faced financial hardships during medical treatments. Besides, there were significant associations between efficiency scores and percentage of the women in the catchment area and the presence of population they in need of social-economic support.

We also observed a strong and significant association between VRS efficiency score and number of hospital beds (capacity). However, there were weak associations between efficiency scores and nationality of catchment populations and other age groups (older sub-groups) and with the number of dispensed prescriptions from the pharmacy department in the hospital. We applied Breusch–Pagan test to assess heteroskedasticity among the variables. The coefficient for this test was 1.43 (*p*-value of 0.157), which revealed the absence of heteroskedasticity.

Tobit regression analysis was employed to relate the technical inefficiency scores to the external variables, while we controlled for the geographic location of the hospitals. The results are presented in Table 4.

**Table 4:** Tobit regression and Two- part model analysis (*N* = 91)

Explanatory variable	Tobit model	Two-part model	
	Coefficient (SE)	1st part	2nd part
		Coefficient (SE)	Coefficient (SE)
Population (n)	−0.548***(0.179)	−12.568*(7.199)	− 0.343 (0.259)
Female (%)	0.030 (0.020)	−0.296 (0.270)	0.016 (0.024)
Non-Saudi (%)	0.029 (0.021)	0.255 (0.295)	0.029 (0.023)
Children (0–5 years)(%)	−0.058**(0.029)	−0.100 (0.264)	0.041 (0.042)
Adults (15–45 years)(%)	0.034***(0.013)	0.145 (0.146)	0.039***(0.014)
Elderly (> 65 years) (%)	0.040**(0.018)	0.213 (0.210)	0.069***(0.021)
Infectious disease (%)	−0.041***(0.015)		
Anti-microbial pres. (n)	0.109 (0.075)	0.472 (0.528)	0.020 (0.062)
Chronic dis. Pres. (n)	−0.222**(0.100)	−0.743 (0.751)	−0.298***(0.109)
Financial hardship (n)	−0.506**(0.197)	−19.054*(11.473)	−0.487**(0.235)
Social support (n)	0.489**(0.193)	10.443* (6.305)	0.495**(0.231)
Region Category			
Central	0.183 (0.506)	16.495 (2621.05)	0.240 (0.567)
North	0.137 (0.545)	9.223 (2621.03)	0.566 (0.615)
South	0.370 (0.496)	11.972 (2621.04)	0.237 (0.564)
West	−0.007 (0.508)	4.765 (2621.02)	−0.023 (0.574)
_Constant	4.165**(2.040)	40.710 (2621.12)	4.325*(2.603)
var (CRS)	0.970 (0.166)		
LR chi2(15)	63.89	85.22	2.17
Prob> chi2	0.00***	0.00***	0.021**
Pseudo R2	0.238	0.846	0.193
Log-likelihood function	−102.285	−7.719	−94.416

A negative coefficient indicated a positive association with CRS, and a positive coefficient meant a negative association with CRS. SE, standard error. Non-Saudi, percent of non-Saudi; Female, the proportion of female; Infectious disease, percentage of infectious diseases, Anti-microbial pres., amount of antimicrobial dispensed medications; Chronic dis. Pres., chronic prescriptions dispensed; Financial hardship, a number of the population faced financial hardship; Social support, number received benefits of social-economic support. * Significant at the 0.10 level, two-tailed test. **Significant at the 0.05 level, two-tailed test. ***Significant at the 0.01 level, two-tailed test

Regarding environmental factors, the number of population (*p* = 0.003) and percentage of children (0–5 years) in the catchment area (*p* = 0.047) were statistically significant and assumed negative signs with technical inefficiency, indicating that the hospitals with more population and more proportion of children in the catchment area have higher efficiency scores. However, the proportions of adults (15–45 years) and elderly (> 65 years) in the catchment populations exhibited a positive and significant association (*p* = 0.009) and (*p* = 0.025) respectively, indicating that sample hospitals with a higher proportion of adults and elderlies were technically inefficient. On the other hand, the percentage of female (*p* = 0.132) and non-Saudi (*p* = 0.161) of the catchment populations had no significant association with the inefficiency scores.

The population with infectious diseases (*p* = 0.007) and the number of chronic medication prescription dispensed from pharmacy departments (*p* = 0.029) were statistically significant with negative associations with technical inefficiency. That indicates that the hospitals that served patients with infectious disease and dispensed more chronic medication prescriptions achieved higher efficiency scores. Whereas, antimicrobial prescriptions show no such association (*p* = 0.151).

The number of populations that faced financial hardship during the treatment was statistically significant with inefficiency scores (*p* = 0.012), with a negative coefficient indicating that more populations with financial hardship in the catchment area experienced higher efficiency scores. Also, the number of populations who received financial support from social administration were statistically significant, with inefficiency scores (*p* = 0.013).

In the first stage of the two-part model, we found that number of population in the catchment area (*p* = 0.081), number of the population faced financial hardship (*p* = 0.097) and those received financial support (*p* = 0.098) were significantly associated with the hospital efficiency. In the second stage of two-part model, the financial hardship cases (*p* = 0.039), financial support (*p* = 0.032) and chronic medication prescriptions (*p* = 0.007), in addition to number of elderly (*p* = 0.001) and adult (*p* = 0.008) population showed significant association with efficiency scores.

Our control variable, i.e., the hospital geographic locations (institutional factor) showed no significant association with the technical inefficiency scores.

## Discussion

This study explained the variations in technical efficiency levels of the public hospitals by the external factors, i.e. institutional and environmental characteristics of the population in the catchment areas of the relevant hospitals and estimated the size of their impact. The empirical investigation is essential in identifying the factors that influence the productivity of these hospitals and in creating evidence for designing the health policies for achieving the optimal level of usage of health resources [31]. However, the assessment of institutional and environmental factors, which often shows significant effects on the efficiency level of public hospitals, have not been analyzed sufficiently in the KSA.

We assessed the effects of the external factors on the technical efficiency of the public hospitals which were affiliated to the MOH in Saudi Arabia. In the previous study, we applied an efficiency analysis of 91 public hospitals using Data Envelopment Analysis (DEA) with input-orientation to estimate the technical efficiency scores [18]. The analysis was based on four inputs and six outputs variables. The input variables were the number of hospital beds, number of physicians, nurses and allied health personnel. Whereas the output variables used were the number of outpatient visits, discharged patients, surgical operations, radiological and laboratory tests and hospital mortality rate [18]. The technical efficiency scores varied dependently on the hospital size in four categories and geographic location of the hospitals in five categories. The average technical efficiency score of the hospitals was 76%. It was also observed that the small-size hospitals and central-region hospitals were relatively more technically efficient than larger hospitals or the hospitals that were in other geographic regions (Table 1) [18].

In this study, the spearman correlation results indicated strong associations between technical inefficiency scores (CRS, VRS, and scale inefficiency) and most of the external factors. Further, the Tobit regression and the Two-part models showed that the environmental and institutional factors had a significant influence on inefficiency scores. We found that hospitals efficiency scores were significantly associated with the population’s density in the catchment area (Coef. -0.548, 95% CI: − 0.904;-0.192). In other words, the hospitals with larger catchment populations had a higher chance to be technically efficient. Similar findings were observed in other studies [14, 32]. The reason of such association could be explained by that the larger population density and relevant various health needs might be the major driver of the demand for healthcare and consequently higher utilization of various health services in public hospitals [33]. This increased utilization in response to higher demand might have increased the health services production from a given hospital (hospital outputs) and resulted in higher efficiency scores according to technical efficiency definition by Farrell in 1957 [34].

On the other hand, the allocation of resources/inputs in public hospitals, which were used to produce hospital outputs, might not have done considering the size of the population in the catchment area but was affected by the hospital size (number of beds). Therefore, there are possibilities of health-resource wastage in hospitals with small catchment population, which could affect the efficiency scores significantly [14]. It was also observed that though the proportion of female and non-Saudis in catchment area showed an association with efficiency scores in the correlation analysis, the Tobit model did not confirm these associations.

The analysis showed that hospitals efficiency scores were associated significantly with the proportion of children in negative coefficient (meaning higher efficiency), but in positive coefficients (meaning lower efficiency) with adults and elderlies; revealing that these age groups had opposite effects on efficiency scores (Tables 3 & 4). Efficient hospitals had a higher proportion of children. It can be argued that these children (0–5 years) might have a higher level of morbidity and a higher need for healthcare, especially in their early years of life and which resulted in more health services utilization in the hospitals [35]. Several types of health services were utilized mainly by children compared to older patients, for instance, immunization services by the outpatient department in the public hospitals. However, we found that public hospitals with a large proportion of adults and elderlies in the catchment area had a higher chance of inefficiency. This finding was in the line of some previous studies in health sectors [26].

The results of the Tobit model indicated a strong association between the proportion of the population with infectious diseases and inefficiency scores (coefficient − 0.041, *p* = 0.007). A similar association was found in both models between efficiency scores and a dispensed prescription for chronic medication (coefficient − 0.222 *p* = 0.029). The findings indicated that public hospitals served more patients with infectious diseases and dispensed more chronic medication prescriptions, leading to higher technical efficiency scores. The possible explanation could be found in the process of treatment of infectious disease patients in the hospitals, meaning that these patients often required acute treatment in a short period, like one visit to an outpatient clinic, followed by a start of the antibiotic course for each patient. On the contrary, the chronic disease patients who needed more comprehensive treatment, but over a more extended period, i.e., several months based on the clinical needs might have consumed a lesser number of services, given health resources, which thus contributed to technical efficiency of the hospitals.

The chronic disease prescriptions were steadily increasing due to the increasing prevalence of incidences, especially diabetes and hypertension, in Saudi Arabia [3, 7]. These common chronic diseases required medication treatment for the entire lifetime of the patients, and thus, prescriptions are dispensed from the pharmacy department in each public hospital. This higher number of dispensed medications for chronic disease was one of the critical hospital outputs in Saudi Arabia, which improved the overall performance of the hospitals.

The number of populations who faced financial hardships during the treatment and those who received financial support from social administration in the catchment area, as indicators of poverty in the catchment area, were associated significantly with inefficiency scores in Tobit and Two-part models. It thus implied that the public hospitals covering a higher proportion of the population in poverty were relatively more efficient than the hospitals with a lower proportion of poor people. Similar findings of other researcher justified that poorer people utilized more services due to free higher access to public hospitals [35]. Further, many literatures argued that poorer people appeared to be more frequently in illness and needed more healthcare services [36]. The findings could be attributable to the fact that public facilities were more utilized by deprived people than the wealthier ones, which facilitated service production compared to the hospitals with a lower proportion of poor people [37, 38].

We suggested that the efficiency in resource allocation should be improved by considering different demographic and socioeconomic indicators as well as the health status of the catchment populations (e.g., population density, poverty, health indicators and services utilization) [32]. The MOH should pay more attention to equality based on different population characteristics when building and amending health policies and planning [39]. It is crucial to ensure the appropriate allocation mechanisms of health resources, and to improve the utilization of health services among the target populations, for securing efficient and equitable health services to achieve universal health coverage [39].

The policymakers should consider the appropriate use of resources within hospitals as well as to re-allocate resources across hospitals, given the findings of the research in public sector efficiency and responding to the health needs of the population [40]. To improve health outcomes, reduce the gap between health care provision, health status and population needs, more attention should be paid to the hospitals that serve high-density populations, more children, a higher percentage of poverty and high incidence of infectious diseases [40]. Also, it is essential to engage the primary health centres for supporting health service provisions, especially in terms of infectious disease control and follow up the chronic disease cases, e.g. diabetic and hypertensive patients, in the region [21]. Such policy and practice initiatives may make the efficient use of health resources to ensuring the maximum value for money, which should contribute significantly towards achieving universal health coverage in the KSA.

It is important to understand the contribution of external factors and population demands on the efficiency of health care services since they significantly influence health care utilization towards efficiency. We encourage the health stakeholders to understand the supply and demand sides of the health care system.

Future research should consider the specific population needs and service profiles of the public hospitals, as well as the impact of need and accessibility on the utilization of health services. Conducting further technical and allocative efficiency research considering environmental and institutional factors specifically for each category (like, based on size and locations) of the hospitals. Further, it is important to indicate the current weaknesses in healthcare production processes and consequently would guide policymakers in potential reforms of health policy and directives.

We faced the challenges of finding data on poverty headcount or the proportion of people living below the poverty line in the hospital catchment areas. Because this information was not available in either the Saudi or the global data sources (e.g. World Bank), we used two variables as the poverty indicators in the hospital area; the number of populations who faced financial hardship during the treatment and number of cases investigated and economically supported by the public social administration.

The number of catchment population referred to the number of populations in the hospital’s area, who were registered in the relevant (generally nearby) public hospitals. The number of catchment population might be inaccurate sometimes and difficult to measure precisely, as patients often referred to the hospitals that were more easily accessible or closer to the patients’ residence rather than those to which they are assigned, especially in a high-density urban area where several hospitals were located in the same city. The borderline between secondary care hospitals and primary centres was often unclear in Saudi Arabia. Due to the absence of a referral system, the patients were practically free to be referred to any service providers, which might result in double-count of the same patients in more than one hospital. Improvement of the current referral health system was crucial to optimize patient health records. Thus, the patient records system should be improved for estimating the optimal levels of health resources that should be in place and prevent wastage of resources in the hospitals. Despite a few limitations, this first study of its kind in the KSA is expected to create strong interest among policymakers, stakeholders, researchers, and academics.

## Conclusions

The findings indicated that the efficiency scores of public hospitals were associated significantly with the population’s density in the catchment area, the proportion of children, proportion of populations in need, infectious diseases cases and the number of dispensed prescriptions. Worldwide, the MOHs should pay more attention to the performance of public hospitals considering the health needs and demographic characteristics of the catchment populations.

Further assessments and research are needed in technical and allocative efficiency in addition to determinants of efficiency, to generate evidence-based knowledge that focuses the causes of inefficiency and challenges in healthcare production in the public sector and to guide the potential reforms of health policies and goals for public hospitals in the KSA and the globe.

In sum, the efficiency in resource utilization and policy planning of resource allocation should consider the different demographic and socioeconomic factors as well as disease conditions and health status of the catchment populations of the hospitals. Understanding about the need for care, access to services and demand for health care is essential for improving the usage of health resources to ensure equitable use of services and better value for money in public hospitals.

## Abbreviations

UHC: Universal health coverage
KSA: Kingdom of Saudi Arabia
MOH: Ministry of health
WHR: World health report
DEA: Data envelopment analysis
CRS: Constant return to scale
VRS: Variable return to scale
SE: Standard error
